# Energy Consumption for Furniture Joints during Drilling in Birch Plywood

**DOI:** 10.3390/polym16081045

**Published:** 2024-04-10

**Authors:** Weronika Pakuła, Barbara Prałat, Zbigniew Potok, Krzysztof Wiaderek, Tomasz Rogoziński

**Affiliations:** Department of Furniture Design, Faculty of Forestry and Wood Technology, Poznań University of Life Sciences, ul. Wojska Polskiego 38/42, 60-627 Poznan, Poland; 70655@student.up.poznan.pl (W.P.); barbara.pralat@up.poznan.pl (B.P.); zbigniew.potok@up.poznan.pl (Z.P.); krzysztof.wiaderek@up.poznan.pl (K.W.)

**Keywords:** furniture joints, drilling, energy consumption

## Abstract

The purpose of this study is to support eco-design ideas and sustainable manufacturing techniques by examining the energy consumption related to drilling holes for different furniture connections. The experimental model is a simple piece of furniture made from birch plywood with three different types of joints. Eccentric joints, confirmat screws, and dowel measurements of energy consumption with a CNC drilling and milling machine show different values for every kind of connector. The energy consumption was measured using a portable power quality analyzer, specifically the PQ-box 150 manufactured by A:Eberle GmbH & Co. KG Nürnberg, Germany. This device likely adheres to industry standards for energy measurement, ensuring accurate and reliable results. The measurement process involved recording energy consumption at different stages of the machining process, allowing for the analysis of specific cutting work and total energy consumption for various joint types. Dowels exhibit the lowest energy consumption at 0.105 Wh for one furniture joint, confirmat screws at 0.127 Wh, while eccentric joints, despite their higher energy consumption (0.173 Wh), offer enhanced transportability and assembly flexibility of a piece of furniture. Specific cutting power for one selected piece of furniture was 227.89 J/mm^3^ for dowels, 190.63 J/mm^3^ for eccentric joints and 261.68 J/mm^3^ for confirmat screws.

## 1. Introduction

Circular economy principles and a product’s environmental impact should be considered from the very beginning of its design. The term “eco-design” refers to the integration of various disciplines, including ecological engineering, ecological restoration, green architecture, and others [1]. Eco-design must consider all aspects of the product’s life cycle, including shipping, machining, waste generation overall, and the product’s end, in addition to materials and aesthetics [2].

The optimization of the production process brings benefits to the environment with an almost immediately achievable goal: reducing the environmental impact of individual stages of material processing by changing various process factors, combined with the development of more advanced technologies and efficient processing methods [3]. The manufacturing sector is leading the way in lowering energy consumption because of the industry’s constant rise in energy, the usage of energy receivers, and the increasing amount of energy used in the production process. This includes the fact that variables influencing energy consumption have an impact on cutting parameters [4]. Over the past fifteen years, many energy consumption models have been published, which is particularly significant when it comes to energy-minimization machining. The relationship between energy consumption and the material removal rate (MRR) in a typical machining operation was initially theorized by Gutowski [5].

Specific cutting energies can be employed as machining-process efficiency indicators in most material removal operations. The energy required to create new surfaces, the energy expended in the primary and secondary deformation zones, and the interfacial friction activities at the tool–workpiece interfaces are all examples of specific cutting energies. During the cutting process, this energy is transferred from the cutting tool to the chip, workpiece, and heat via the cutter rake and flank surfaces. The ratio of the volume of workpieces to the energy consumed during machining operations is known as the specific cutting energy. The following factors affect the specific cutting energy: growl production speed, cutting force, and cutting speed [6]. Furthermore, the energy efficiency of the machining process is often determined using that specific cutting energy [7]. To achieve process-level energy efficiency in processing, it is essential to comprehend the impact of material properties and process variables on the required cutting energy factor [8]. In grinding processes, the selection of cutting conditions can result in notable alterations and an increased specific cutting energy [9].

When drilling holes, energy consumption is the most important parameter, but the quality of holes made for selected furniture joints is equally essential. The degree of dimensional accuracy in machining results in a higher vulnerability to errors during the assembly of furniture components. Because of the characteristics of the workpiece, the drill tip may slip during the initial drilling phase, potentially leading to inaccurate machining [10]. When compared to the holes created in two adjacent layers of veneer, the holes created in the adhesive layer displayed deviations that were roughly twice as large. The errors in the centers’ positions did not significantly correlate with the deviations of the holes’ axis angles. Wood shrinkage and swelling have an impact on how effectively furniture pieces work together. Thus, they should be considered while designing. This study investigated how moisture variations affected the drilled holes’ effective diameter [11]. The intricate variations in hole shape caused by moisture are as follows: depending on the depth and wood grain pattern, the holes ovalized with varying ranges, and at the bottom, their diameters were slightly enlarged [12].

In other industries, there has also been an attempt to improve the efficiency of cutting-energy utilization. The aircraft industry makes considerable use of metal materials, particularly multilayer stacks made of CFRP (carbon fiber-reinforced polymer) and Ti6Al4V (Titanium-64, or Grade 5 Titanium Alloy), because of their high specific stiffness, outstanding corrosion resistance, and exceptional structural efficiency. In CFRP/Ti6Al4V stack drilling, a comparative analysis between various cooling conditions was conducted. To ascertain the impact of various cooling techniques on the torque, specific cutting energy, and surface morphology of the processed composite material during CFRP/Ti6Al4V stacks, a series of drilling tests were conducted under dry circumstances and minimum-quantity lubrication. Technical advice for choosing a cooling technique and enhancing energy efficiency in the titanium-composite process machining operations can be obtained from this work [13]. Other research on energy modeling and processing visualization have been acknowledged as useful and efficient methods for identifying areas for energy savings and enhancing energy efficiency. The examination focused on the study’s drilling visualization goals and energy modeling. Drilling procedures revealed that the suggested drilling energy model had an average forecast accuracy of 96.2%. Additionally, the results demonstrated a 12.6% increase in energy efficiency and 7417.8 J of energy savings. The suggested approach helped the drilling process save energy by guaranteeing the energy model’s high accuracy, identifying possible energy savings, and enhancing energy efficiency [14].

In the wood industry, cutting forces are also examined. The impact of introducing SBR gum granulate (Styrene-Butadiene-Rubber gum granulate) on particleboard machinability was examined in a study, with a particular emphasis on cutting forces during drilling. Various formulations, including 0% to 30% SBR, were investigated. The tests, which were carried out on a CNC machine using a 10 mm diameter polycrystalline diamond drill, showed that adding more SBR significantly improved machinability. Relative machinability indicators based on axial force (MIF) and torque (MIM), which show improved machinability, particularly at increasing SBR content, are included in the study. The results demonstrate how SBR gum granulation reduces cutting forces during drilling, which raises the possibility that it could enhance wood-based composite materials used in machining [15]. The analysis of cutting forces during the thermally modified ash-wood drilling process was the main goal of another study. Two sets of workpieces were used in the experiment; one set underwent heat alteration, while the other did not. Both sets of through-holes were drilled, and piezoelectric sensors were used to measure the thrust force and torque data. When thermally changed ash wood was drilled, the results showed a statistically significant increase in thrust force and a statistically significant decrease in torque when compared to the unmodified equivalent. Regarding yearly rings, the tool feed orientation did not have a statistically significant effect on cutting forces. In conclusion, it was discovered that thermally altering ash wood changed the cutting forces, particularly by decreasing torque and raising thrust force, throughout the drilling operation [16].

According to a not fully specified definition, cutting can be considered as changing the density of the workpiece by the mechanical removal of material. Parameters such as cutting speed, feed per revolution, cutting depth, and cross-sectional area of the cut layer are needed to determine energy consumption [17]. The cutting force can be calculated using the above parameters, which allows for energy estimation. As of 2016, Poland was 30.3% dependent on the import of energy raw materials [18]. A piece of furniture with the same structure and function in large-scale production may have different costs if you take a closer look at it, considering the type of connection. Holes for appropriate connections may differ in drilling depth, execution time, and, therefore, the tool’s operating time, diameter, and quantity [19]. The study presented herein addresses a critical gap in the existing body of scientific literature concerning the energy consumption associated with drilling holes for furniture joints. Surprisingly, there remains a paucity of comprehensive research in this domain, highlighting the significance of this paper for both academic researchers and the furniture industry. By delving into the energy usage during the machining process for various furniture connections, this study not only fills an important knowledge void but also offers valuable insights into eco-design practices and sustainable manufacturing techniques. The findings have the potential to inform future research endeavors and guide decision-making processes within the furniture sector, ultimately advancing the pursuit of environmentally friendly production methods. For a furniture designer, how much energy is consumed when drilling holes should be known. If the designer wants their product to be as environmentally friendly as possible, energy consumption when drilling holes for furniture joints should be as low as possible with the assumed functionality. Therefore, the purpose of this work is to verify how selected furniture connections influence energy consumption for drilling holes.

## 2. Materials and Methods

### 2.1. Furniture Construction

For the study, a piece of furniture with a simple structure was designed. The shelf was designed to use three different furniture joints while maintaining the same type of material, shape, and usability. The furniture design consists of two wreaths, two sides, a vertical partition, and two horizontal partitions. The furniture dimensions are: 800 mm length, 1000 mm height, and 400 mm depth. Moreover, 15 mm thick birch *Betula pendula* plywood (Paged Pisz Sp. z o.o., Pisz Poland) was used to make the furniture.

### 2.2. Stiffness Test

The stiffness assessment of birch plywood was performed following the PN–EN 310 [20].

The load was applied to the canter of the sample supported by two supports. The sample dimensions were 350 mm × 50 mm × 15 mm. The marking was performed based on 10 plywood samples with external layers arranged longitudinally, and 10 plywood samples with external layers arranged transversely. The elastic modulus was calculated from the sample deflection in the linear range of deflection and force dependence.

The furniture body stiffness was calculated from the formula: *G*—Kirchhoff modulus, *d*—plate thickness, *l*_1,2_—dimensions of the *i*-th plate, and α—α coefficient, calculated differently depending on the element.

(1) $$k=\sum_{i=1}^{n}\frac{G_{i}{\mathrm{d}}_{i}^{3}}{3{\left(l_{1}l_{2}\right)}_{i}}\alpha_{i}^{2}$$

The global stiffness of the tested structure exceeded the values recommended by standards by more than twice, which allowed for the adoption of a solution in engineering practice for furniture subject to higher loads, such as library shelves. Regardless of the selected connection, the global stiffness remained unchanged.

### 2.3. Numerical Study

**Figure 1:**
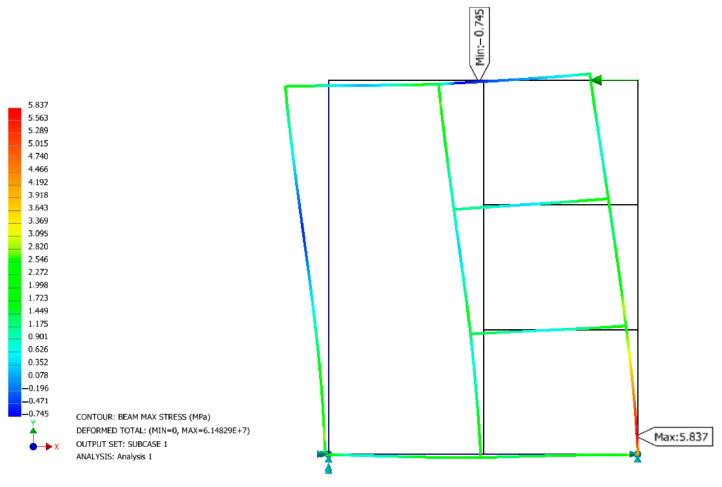
Inventor Nastran tension of the structure when a force was applied.

A numerical study was performed in the Inventor Nastran 2024 program, as shown in Figure 1. The numerical analysis was carried out to determine the correctness of the structure in terms of stiffness and to determine the maximum stresses regardless of the type of furniture joints used. A simplified structure was made reflecting the designed product by entering the corresponding parameters for the selected material. In the case of Poisson’s ratio, it was taken from the article “Theory of elasticity of an anisotropic body”. The calculations confirmed the observations for constant material values. The stiffness *K* [N/mm] depends on the dimensions of the individual structural elements of the furniture. In the case of the analyzed structure, the furniture elements press on each other, exerting contact stresses of no more than 0.6 MPa.

### 2.4. Types of Joints

**Figure 2:**
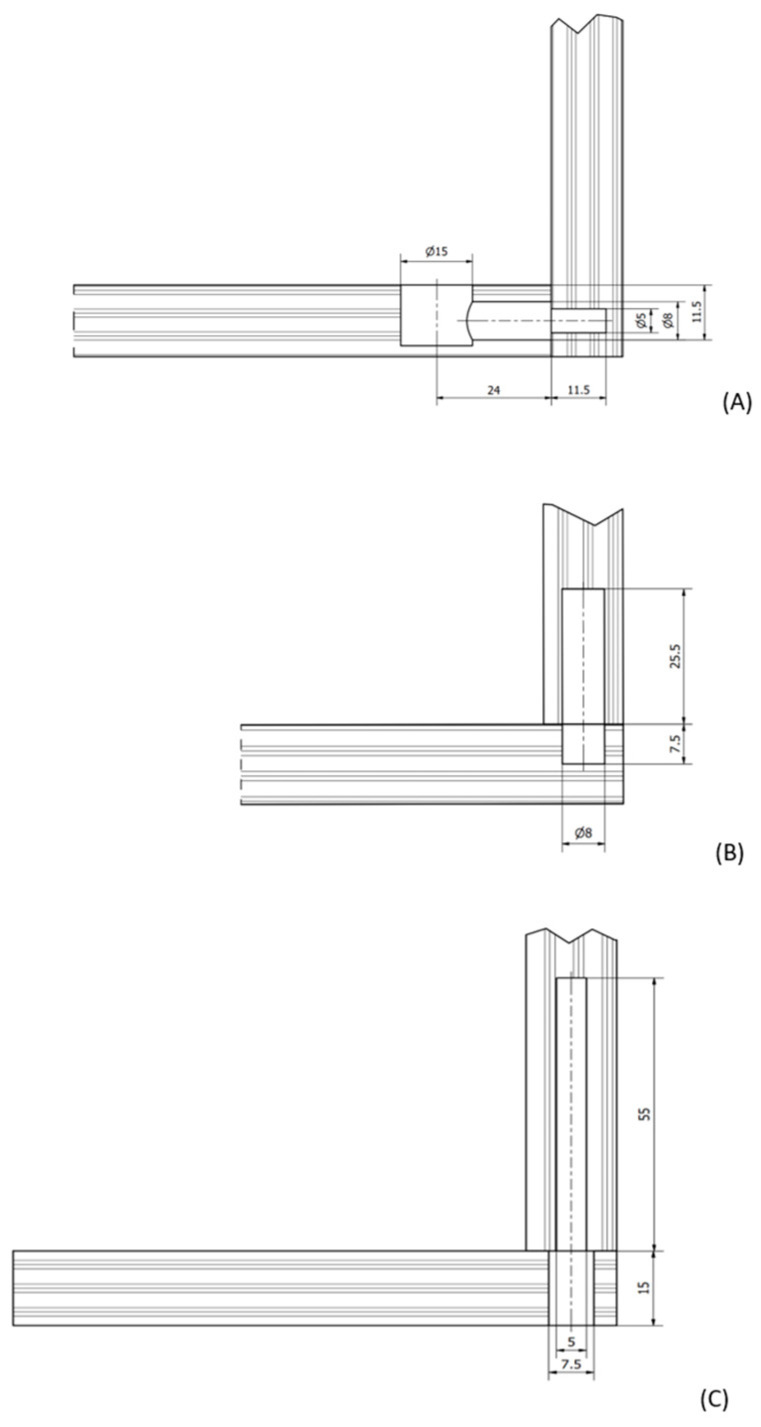
(**A**): drilling diagram for an eccentric joint (three holes); (**B**): drilling diagram for a confirmat screw (two holes); (**C**): drilling diagram for a dowel connector (two holes). Measurements are mm.

**Figure 3:**
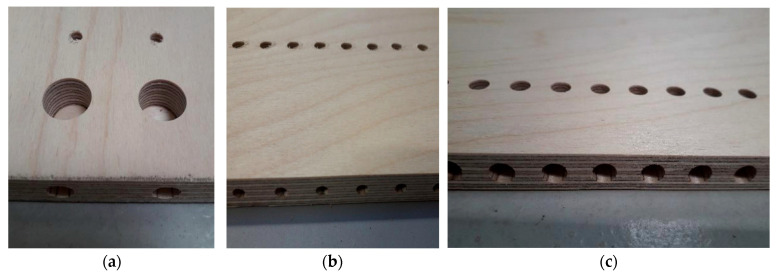
(**a**): drilled holes for an eccentric joint; (**b**): drilled holes for a confirmat screw (two holes); (**c**): drilled holes for a dowel connector (two holes).

Three types of joints (Häfele, Nagold, Germany) were selected and tested. Dowel joints use glue as a permanent connection. The second type was the confirmat screw, which is used as a conditionally detachable connection, enabling the furniture to be unscrewed and reassembled. After reassembling the furniture, it does not have the same strength due to losses in the material caused by the opening of the furniture connection. The last joint considered for testing is the eccentric joint, as a detachable joint. Drilling was performed for 30 furniture joints of each type, as shown in Figure 2. In Figure 3, drilled holes for each joint are shown.

### 2.5. Tools and Machine Used

To make holes for the pins, drills with a diameter of 8 mm were used: a screw drill with a centering spike and cutters that was non-uniform, single, two-edged, and single-stage, with a cylindrical shank; an ordinary drill with a diameter of 8 mm; and a screw drill with a centering spike and cutters, which was non-uniform, single, two-edged, and single-stage, with a cylindrical shank. To make the holes for the eccentrics, drills with a diameter of 5 mm were used: screw drills with a centering spike and cutters that were non-uniform, single, two-point, and single-stage, with a cylindrical shank, and ordinary drills with a diameter of 15 mm that were straight with a centering spike and cutters, non-uniform, and had a cylindrical shank. Drills with a diameter of 8 mm were used, including screw drills with a centering spike and cutters that were ordinary, non-uniform, single, two-edged, and single-stage, with a cylindrical shank, For drilling confirmats screws, drills with a diameter of 5 mm were used, including screw drills with a centering spike and cutters that were ordinary, uniform, single, two-point, single-stage, and had a cylindrical shank, as well as drills with 7 mm diameter screws with a centering spike that were uniform, single, two-point, and single-stage, with a cylindrical, ordinary shank. The drills were provided by ITA Tools Sp. z o.o. (Cracow, Poland). A CNC drilling and milling machine was used for testing Pass-through CNC creator 950 by Felder Group (Hall in Tirol, Austria) with a feed of 2 m/min, and revolutions of 6000 rpm. The energy consumption was measured using a portable power quality analyzer PQ-box 150 (manufacturer: A:Eberle GmbH & Co. KG, Nürnberg, Germany). This type of quality meter was used for the first time with good results for this purpose. So, this is a documentation of the new and effective method for measuring energy consumption during individual operations in the processing of wood-based materials.

### 2.6. Tests Performed

**Figure 4:**
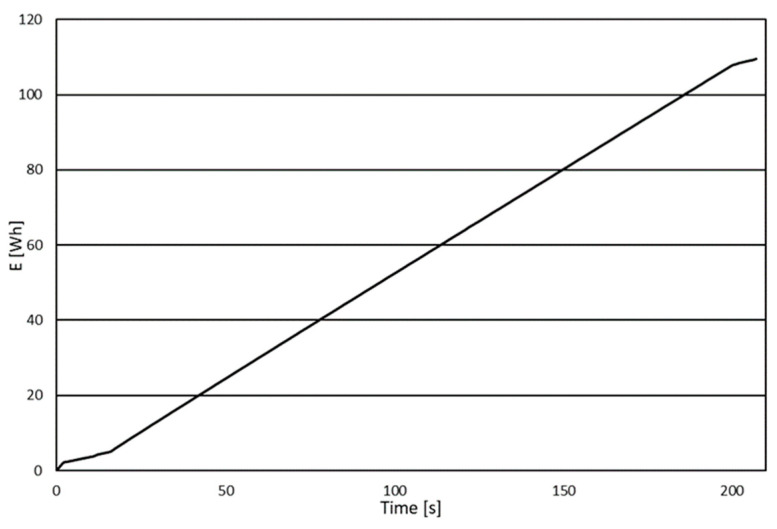
A sample chart showing the total energy consumed obtained from the analyzer.

For each of the tested joints, after preheating the machine, the first stage involved performing so-called zero tests. Their aim was to determine the amount of energy used to operate the machine during the machining program (e.g., drive and movement of the drill, material movement, or computer operation) without the energy used during actual cutting. This was achieved by “tricking” the machine by using a smaller workpiece so that the machine executed the correct machining program, but the tool had no contact with the material. Three zero tests were performed for each type of connection as Test 0, and the average of these measurements, denoted *E0*, was used for calculations. The next stage involved conducting actual tests, where the same program ran, but this time, the tool drilled holes in the material. Before running the CNC program each time, the energy consumption was measured. The recording stopped shortly after the machine finished executing the program. The energy analyzer determined the amount of energy consumed by the machine during operation. For every joint, three tests were run (*j* = 1, 2, 3). A sample chart showing the total energy consumed obtained from the analyzer is presented in Figure 4.

**Figure 5:**
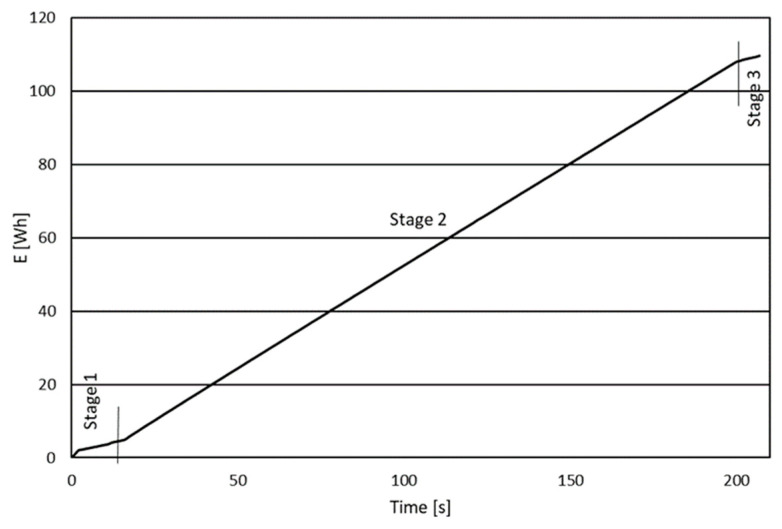
Range of each step of the energy consumption curve.

The results obtained in this way needed to be analyzed. The energy consumption curve can be divided into three stages depending on the machine’s operation and the power quality analyzer’s activity. The first stage was the time between starting the recording on the analyzer and starting the program on the machine. The second stage involved the actual execution of the machining program, while the last stage was the time between completing the program execution and stopping the recording in the energy analyzer. The ranges of each stage are presented in Figure 5

**Figure 6:**
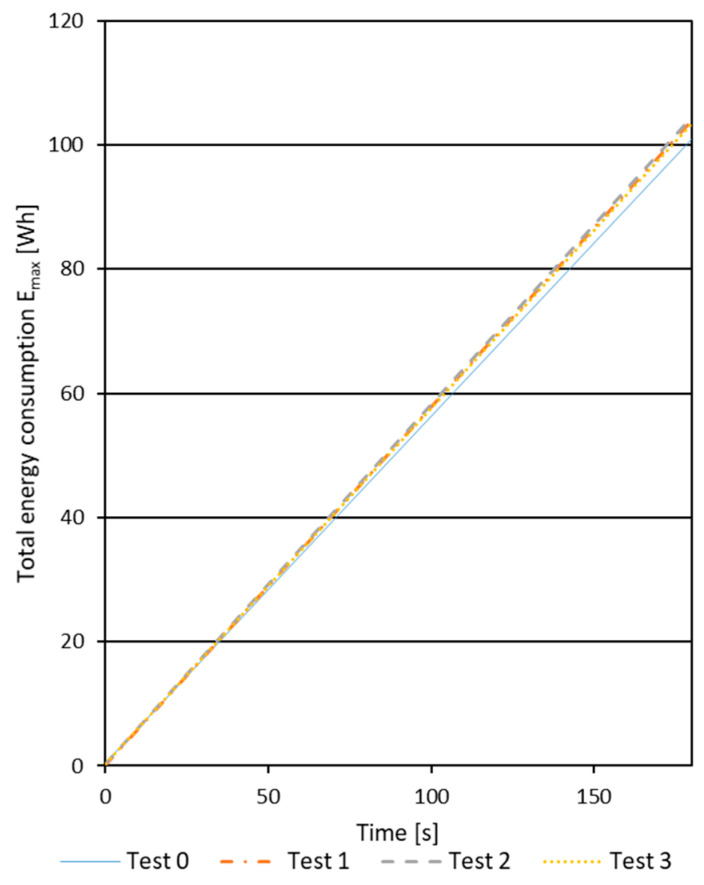
Sample results for the dowel joint after excluding stage 2.

Key to this analysis is stage 2, during which the machining of the workpiece was carried out. Based on the results obtained from the power quality analyzer, charts were created only for stage 2. The results from the analyzer were converted by creating charts for stage 2, in which the energy determined by the analyzer was reduced by the value occurring at the transition from stage 1 to stage 2. For each connection, stage 2 for both the zero tests and the actual tests had to last the same amount of time, namely 184 s for the dowel, 220 s for the confirmat screws, and 280 s for the eccentric joints. Sample results for the dowel connection after excluding stage 2 are presented in Figure 6.

The results obtained in this way could be used to calculate the actual cutting energy during the connection and the total energy required to make the joint. The total energy consumption to make the joint can be calculated from Equation (2):

(2) $$E_{p}=\frac{E_{max}}{30}$$

*E_p_*—total energy consumption to make the joint [Wh].

*E_max_*—total energy consumption for executing the machining program for all holes to make a joint.

The specific cutting work (SCW) was calculated based on Equation (3):

(3) $$SCW=\frac{E_{jmax}-E_{0max}}{\sum_{n=1}^{i}V_{n}}$$

*SCW*—specifying cutting work, J/mm^3^.

*E_jmax_*—total energy consumption during joint execution.

*E*_0*max*_—total energy consumption during the zero test.

*V_n_*—the volume of holes needed to make the connection.

*i*—number of holes depending on the connection.

*j*—test number.

Because three tests were performed on each joint, arithmetic averages for these three test results of the SCW were calculated. A Student’s T-test was performed, and the standard deviation was determined. The data were collected in Table 1, Table 2, Table 3, Table 4, Table 5 and Table 6.

## 3. Results and Discussion

### 3.1. Energy Consumption

The experimental data of energy measurements and calculated results of energy consumption were collected in Table 1, Table 2, Table 3 and Table 4.

### 3.2. Discussion

The average energy consumption needed to drill holes for 30 dowels (*E*_1*max*_*–E*_0*max*_) is 3.169 Wh, and the energy consumption needed to drill holes for one furniture connection (*E_p_*) is 3.537 Wh, as shown in Table 1. The average energy consumption needed to drill holes for 30 eccentric joints (*E*_1*max*_*–E*_0*max*_) is 5.210 Wh, as shown in Table 2. The energy consumption needed to drill holes for one eccentric joint (*E_p_*) was, on average, 5.538 Wh, which is 0.328 Wh more than the energy consumed for drilling one dowel. The energy consumption needed to drill holes for 30 furniture connections (*E*_1*max*_*–E*_0*max*_) is 3.825 Wh, while it was 3.238 Wh for drilling for one connection, as shown in Table 3. Energy data for all drilling tests were calculated for one furniture joint of each type, and their averages were also summarized. Comparing the average energy consumption needed for drilling holes for one furniture joint (*E_p_*), the least energy is consumed while drilling for a confirmat screw (3.238 Wh), and the most energy was consumed while drilling for an eccentric joint (5.538 Wh). The difference between average energy consumption for dowels and confirmat screws and between eccentric joints and other selected furniture joints is smaller because only two holes are needed for dowels and confirmat screws and three are required for eccentric joints. A summary of energy consumption for one selected furniture connection (*E_p_*) is shown in Table 4. A Student’s t-test was performed, and the result was statistically significant. If there were no true difference between the groups, there would be very little chance of producing such a significant difference. As a result, we reject the null hypothesis and support the alternative hypothesis, which implies that the mean values of dowels and confirmat screws differ from one another. Because of the statistical significance of this difference, we may be certain that the results represent genuine group differences rather than just random fluctuations in the data. The standard deviation for one selected furniture connection for dowels and confirmat screws is 0.02; for eccentric joints, this figure is 0.03.

The statistical analysis conducted through t-tests offered valuable insights into the energy consumption disparities among the examined furniture joints. Firstly, the comparison between eccentric joints and confirmat screws revealed a statistically significant difference (*p*-value = 0.003958847), underscoring the distinct energy utilization patterns of these joint types. Additionally, the t-test conducted between dowels and eccentric joints also demonstrated a statistically significant disparity (*p*-value = 0.01477), highlighting differing energy consumption profiles between these two types of joints. Conversely, the t-test between dowels and confirmat screws yielded a non-significant result (*p*-value = 0.6490413), indicating a lack of compelling evidence to suggest a substantial discrepancy in energy consumption between these joint variants. These statistical findings complement the experimental measurements, shedding light on the relative energy efficiency of different furniture connectors and supporting informed decision-making in eco-design and sustainable manufacturing endeavors.

### 3.3. Specific Cutting Work

The data for calculation and calculated results of specific cutting work were collected in Table 5 and Table 6.

The volume of the selected material for drilling holes for one furniture joint was 1658.77 mm^3^ for the dowel, 3299.43 mm^3^ for the eccentric joints, and 261.68 mm^3^ for the confirmat screws, as shown in Table 5. Specific cutting work for one selected piece of furniture was 227.89 J/mm^3^ for dowels, 190.63 J/mm^3^ for eccentric joints^,^ and 26.68 J/mm^3^ for confirmat screws, as shown in Table 6. According to the physics of cutting, the confirmat screws have the smallest hole diameter and a large depth, which makes it most difficult for chips to escape during cutting, compared to a dowel or eccentric joint. It also has the highest specification cutting work rate.

The conducted research unveils a pioneering method for precisely determining specific cutting work. Leveraging the PQ-box 150 energy analyzer, a direct measurement of energy consumption during wood material cutting was conducted. By integrating the outcomes of these measurements with the volume of the resulting hole, the actual cutting work was calculated. In contrast to previous approaches reliant on measuring cutting forces to calculate cutting work, this methodology represents a significant advancement. Previous studies primarily employed rudimentary knives featuring a single cutting surface [21,22,23,24,25]. However, this novel approach enables the determination of appropriate cutting work across various machining tools.

## 4. Conclusions

The above data show that connecting furniture with dowels consumes the least energy but is the least user-friendly as it does not allow for disassembly and reassembly or easy transport. Despite higher energy consumption than dowels, eccentric joints allow furniture to be transported more conveniently and easily than dowels and self-assembly. Unfortunately, each time the furniture is unfolded again, it loses its strength due to the tearing of the material caused by the removal of the confirmat screw. The biggest energy consumption of drilling holes for one furniture connection, and at the same time the most durable and best in terms of technology and use, is for the eccentric joint, which allows the user to assemble the furniture independently and easily disassemble and transport it, without compromising the durability and strength of the furniture. Unfortunately, preparing the best furniture requires more energy due to the number of holes needed and their size. It can be concluded that increasing the usability of a piece of furniture increases the use of technology and, consequently, energy consumption. This study provides insightful information on the energy usage of various furniture joints, which is helpful for eco-design and sustainable manufacturing techniques. Our understanding will grow because of increased research and the constant observation of technical advancements, which will also direct the furniture sector towards more environmentally friendly production practices.

## Figures and Tables

**Table 1:** The energy consumption for dowels.

	Test 0	Test 1 (j = 1)	Test 2 (j = 2)	Test 3 (j = 3)	Average of Tests	Standard Deviation
Wh						
Total energy consumption *E_max_*	102.950	106.150	106.659	105.551	106.120	0.55
The energy consumption needed to drill holes for 30 furniture connections *E_jmax_–E*_0*max*_	-	3.199	3.707	2.6	3.169	0.55
The energy consumption needed to drill holes for one furniture connection *E_p_*	-	3.538	3.555	3.518	3.537	0.02

**Table 2:** The energy consumption for eccentric joints.

	Test 0	Test 1 (j = 1)	Test 2 (j = 2)	Test 3 (j = 3)	Average of Tests	Standard Deviation
Wh						
Total energy consumption *E_max_*	160.964	166.779	165.063	166.682	166.174	0.96
The energy consumption needed to drill holes for 30 furniture connections *E_jmax_–E*_0*max*_	-	5.815	4.099	5.718	5.210	0.96
The energy consumption to drill holes for one furniture joint *E_p_*	-	5.558	5.501	5.555	5.538	0.03

**Table 3:** The energy consumption confirmat screws.

	Test 0	Test 1 (j = 1)	Test 2 (j = 2)	Test 3 (j = 3)	Average of Tests	Standard Deviation
Wh						
Total energy consumption *E_max_*	93.305	97.598	96.424	97.370	97.131	0.62
The energy consumption needed to drill holes for 30 furniture connections *E*_1*max*_*–E*_0*max*_	-	4.292	3.119	4.064	3.825	0.62
The energy consumption needed to drill holes for one furniture connection *E_p_*	-	3.253	3.214	3.246	3.238	0.02

**Table 4:** Summary of energy consumption for one selected furniture connection E_p_.

	Test 1	Test 2	Test 3	Average of Tests	Standard Deviation
Wh					
Dowels	3.538	3.555	3.518	3.537	0.02
Eccentric joints	5.558	5.501	5.555	5.538	0.03
Confirmat screws	3.253	3.214	3.246	3.238	0.02

**Table 5:** Volume for each furniture joint.

	V₁	V₂	V₃	Total V
mm^3^				
Dowels	377.00	1281.77	-	1658.77
Eccentric joints	225.80	829.37	2244.26	3299.43
Confirmat screws	1079.91	662.68	-	1742.59

**Table 6:** Specifying cutting work.

	Test 1	Test 2	Test 3	Average
J/mm^3^				
Dowels	230.05	266.46	186.89	227.89
Eccentric joints	210.63	148.51	207.31	190.63
Confirmat screws	295.54	212.90	278.90	261.68

## Data Availability

Data is contained within the article.

## References

[1] Burchart D. (2010). Ekoprojektowanie—Holistyczne podejście do projektowania. Probl. Ekol..

[2] Zhang Q.M., Zhang W.M. (2013). Material Election and Ecological Design for Furniture Products Based on the Principles of Green Manufacturing. Adv. Mater. Res..

[3] Jayal A.D., Balaji A.K. (2007). On a Process Modeling Framework for Sustainable Manufacturing: A Machining Perspective. Volume 15: Sustainable Products and Processes.

[4] Wang Q., Zhang D., Chen B., Zhang Y., Wu B. (2019). Energy Consumption Model for Drilling Processes Based on Cutting Force. Appl. Sci..

[5] Gutowski T.G., Branham M.S., Dahmus J.B., Jones A.J., Thiriez A., Sekulic D.P. (2009). Thermodynamic Analysis of Resources Used in Manufacturing Processes. Environ. Sci. Technol..

[6] Teimouri R., Amini S., Lotfi M., Alinaghian M. (2019). Sustainable drilling process of 1045 steel plates regarding minimum energy consumption and desired work quality. Int. J. Lightweight Mater. Manuf..

[7] Rahim E.A., Rahim A.A., Ibrahim M.R., Mohid Z. (2016). Experimental Investigation of Supercritical Carbon Dioxide (SCCO2) Performance as a Sustainable Cooling Technique. Procedia CIRP.

[8] Rajemi M.F., Mativenga P.T., Aramcharoen A. (2010). Sustainable machining: Selection of optimum turning conditions based on minimum energy considerations. J. Clean. Prod..

[9] Boothroyd G. (1988). Fundamentals of Metal Machining and Machine Tools.

[10] Sydor M., Rogoziński T., Stuper-Szablewska K., Starczewski K. (2019). The accuracy of holes drilled in the side surface of plywood. BioResources.

[11] Sydor M., Majka J., Langová N. (2021). Effective Diameters of Drilled Holes in Pinewood in Response to Changes in Relative Humidity. BioResources.

[12] Sydor M., Majka J., Rychlik M., Turbański W. (2023). Application of 3D Scanning Method to Assess Mounting Holes’ Shape Instability of Pinewood. Materials.

[13] Ji M., Xu J., Chen M., Mansori M.E. (2020). Effects of Different Cooling Methods on the Specific Energy Consumption when Drilling CFRP/Ti6Al4V Stacks. Procedia Manuf..

[14] Jia S., Cai W., Liu C., Zhang Z., Bai S., Wang Q., Li S., Hu L. (2021). Energy modeling and visualization analysis method of drilling processes in the manufacturing industry. Energy.

[15] Wilkowski J.A., Kozub W.O., Borysiuk P.I., Rousek M.I., Czarniak P.A., Gorski J.A., Podziewski P.I., Szymanowski K.A. (2014). Machinability of particleboards bonded with SBR gum granulate. Ann. Wars. Univ. Life Sci. SGGW For. Wood Technol..

[16] Wilkowski J., Grześkiewicz M., Czarniak P., Wojtoń M. (2011). Cutting forces during drilling of thermally modified ash wood. Ann. Wars. Univ. Life Sci. SGGW For. Wood Technol..

[17] Wilkowski J., Czarniak P., Górski J., Jabłoński M., Podziewski P., Szymanowski K. (2014). Taguchi Method Optimization of the Total Power Consumption During Chipboard Cutting with the Panel Saw Machine. Rieskové Beztrieskové Obrábanie Dreva.

[18] Braun J. (2018). Bezpieczeństwo energetyczne jako dobro publiczne—Miary i czynniki wpływające na jego poziom. Stud. Ekon..

[19] Saloni D.E., Lemaster R.L., Jakson S.D. (2005). Abrasive Machining Process Characterization on Material Removal Rate, Final Surface Texture, and Power Consumption for Wood—ProQuest. https://www.proquest.com/openview/a99e8550a54ea7cb1554f55652a2cc6c/1?pq-origsite=gscholar&cbl=25222.

[20] (1994). Wood-Based Panels–Determination of Modulus of Elasticity in Bending and of Bending Strength.

[21] Atkins A.G., Vincent J.F.V. (1984). An instrumented microtone for improved histological sections and the measurement of fracture toughness. J. Mater. Sci. Lett..

[22] Huang X., Jeronimidis G., Vincent J.F.V., Spatz H.C.H., Speck T. (2000). The Instrumented Micro-Tome Cutting Tests on Wood from Transgenic Plants with Modified Lignification.

[23] Kowaluk G., Dziurka D., Beer P., Sinn G., Tschegg S. (2004). Influence of particleboards production parameters on work of fracture and work of chips formation during cutting. Electron. J. Pol. Agric. Univ. Wood Technol..

[24] Beer P., Kowaluk G., Sinn G., Dziurka D. Mechanical properties of particleboards induce cutting quality. Proceedings of the 17th International Wood Machining Seminar.

[25] Kowaluk G. (2005). Analyzing of Cutting Work due to Quality Machining off Chosen Laminated Chipboards. Ph.D. Thesis.

